# Association of Maternal Use of Triptans During Pregnancy With Risk of Attention-Deficit/Hyperactivity Disorder in Offspring

**DOI:** 10.1001/jamanetworkopen.2022.15333

**Published:** 2022-06-03

**Authors:** Gerd Marie Harris, Mollie Wood, Eivind Ystrom, Hedvig Nordeng

**Affiliations:** 1 Pharmacoepidemiology and Drug Safety Research Group, Department of Pharmacy, PharmaTox Research Initiative, Faculty of Mathematics and Natural Sciences, University of Oslo, Oslo, Norway; 2 Department of Epidemiology, Gillings School of Global Public Health, University of North Carolina at Chapel Hill; 3 Department of Mental Disorders, Norwegian Institute of Public Health, Oslo, Norway; 4 PROMENTA Research Center, Department of Psychology, University of Oslo, Oslo, Norway; 5 Department of Child Health and Development, Norwegian Institute of Public Health, Oslo, Norway

## Abstract

**Importance:**

Triptans are commonly used in the treatment of migraine. Prenatal exposure to triptans may be associated with adverse fetal neurodevelopment; however, there is limited information about the long-term safety of triptan use during pregnancy.

**Objective:**

To examine the association between maternal use of triptans during pregnancy and diagnosis and symptoms of attention-deficit/hyperactivity disorder (ADHD) among offspring.

**Design, Setting, and Participants:**

This study used data from the Norwegian Mother, Father and Child Cohort Study (recruitment 1999-2008), linked to national health registries. Live-born singleton children born to women with migraine before or during pregnancy were included. Two analytic samples were defined: one to assess ADHD diagnosis and one to assess ADHD symptoms. Data were analyzed from May 1 to November 30, 2021.

**Exposure:**

Maternal self-report of triptan use during pregnancy. Exposed children were compared with 2 groups of unexposed children whose mothers reported migraine (1) during pregnancy and (2) before pregnancy only.

**Main Outcomes and Measures:**

An ADHD diagnosis was defined as diagnosis of hyperkinetic disorder or receipt of dispensed ADHD medication. Symptoms of ADHD at 5 years were measured by the Conners’ Parent Rating Scale, where a higher score indicates more symptoms of ADHD. Cox proportional hazards regression models and generalized linear models with inverse probability weights were used to estimate weighted hazard ratios (HRs) and standardized mean differences, respectively, with 95% CIs.

**Results:**

The ADHD diagnosis sample comprised 10 167 children (mean [SD] maternal age, 30.2 [4.6] years; 5231 boys [51.5%]), and the ADHD symptoms sample comprised 4367 children (mean [SD] maternal age, 30.6 [4.4] years; 2191 boys [50.2%]). Children were followed up for a mean (SD) of 10.6 (2.2) years. Children with prenatal triptan exposure had no increased risk of ADHD diagnosis compared with unexposed children whose mothers had migraine during pregnancy (weighted HR, 1.16; 95% CI, 0.78-1.74) and compared with unexposed children whose mothers had migraine only before pregnancy (weighted HR, 1.28; 95% CI, 0.84-1.94). There were no differences in ADHD symptom scores between exposed and unexposed children.

**Conclusions and Relevance:**

The findings of this study suggest that there is no increased risk of ADHD among offspring associated with prenatal exposure to triptans.

## Key Points

**Question:**

Is prenatal exposure to triptans associated with an increased risk of attention-deficit/hyperactivity disorder (ADHD) in offspring?

**Findings:**

This population-based cohort study of 10 167 children found no associations between prenatal exposure to triptans and ADHD diagnosis or ADHD symptoms among offspring.

**Meaning:**

These findings suggest that there is no increased risk of ADHD in offspring associated with prenatal exposure to triptans.

## Teaser

This cohort study uses data from the Norwegian Mother, Father and Child Cohort Study to examine the association between maternal use of triptans during pregnancy and diagnosis and symptoms of attention-deficit/hyperactivity disorder (ADHD) among offspring.

## Introduction

Triptans are the most commonly used prescription medications for acute treatment of migraine, which is a chronic neurologic disorder characterized by moderate to severe headache in combination with other symptoms.^1^ Migraine is the second leading cause of years lived with disability, and it is most prevalent among women of reproductive age, with a peak prevalence of more than 25%.^2^ Information about the safety of triptans during pregnancy is limited, particularly for long-term outcomes, such as child neurodevelopment. Prevalence estimates of triptan use during pregnancy among women with migraine vary between 9% and 25%.^3,4,5^ Many women with migraine discontinue use of triptans during pregnancy or switch to acetaminophen,^6,7^ which is in line with treatment recommendations.^8^ However, a substantial proportion of these women report high pain intensity.^6^ Moreover, untreated maternal migraine has been associated with several pregnancy complications.^9^

Studies of prenatal exposure to triptans during pregnancy have not found an association with increased risks of malformations.^10^ Some studies found increased neurodevelopmental problems in exposed offspring compared with migraine controls, mainly among younger children.^11,12^ These associations have not been observed for preschool-aged children.^13^ Previous studies have mainly used parent-reported outcomes, and there is a need for research on diagnoses of neurodevelopmental problems, such as attention-deficit/hyperactivity disorder (ADHD). Attention-deficit/hyperactivity disorder is one of the most common behavioral disorders in childhood, with a worldwide prevalence of 5%.^14^

The aim of this study was to investigate the association between prenatal exposure to triptans and ADHD among offspring. Specifically, we aimed to investigate both ADHD diagnosis and ADHD symptoms; the latter was investigated so that we could identify children with problems who do not meet the diagnostic criteria.^15^

## Methods

### Data Sources

This study used data from the Norwegian Mother, Father and Child Cohort Study (MoBa), linked to the Medical Birth Registry of Norway, the Norwegian Patient Registry (NPR), and the Norwegian Prescription Database using the mother’s personal identification number. eFigure 1 in the Supplement shows an overview of the time coverage of the data sources. MoBa is a population-based pregnancy cohort study conducted by the Norwegian Institute of Public Health.^16^ All pregnant women who could speak Norwegian were invited to participate in the study from 1999 to 2008, and for 41% of the pregnancies the women provided written consent to participate.^17^ The cohort includes 114 500 children, 95 200 mothers, and 75 200 fathers. An overview of all questionnaires in MoBa can be found at the Norwegian Institute of Public Health webpage.^18^ The present study used information from questionnaire 1 (Q1; sent out in gestational weeks 15-17), Q3 (sent out in gestational week 30), Q4 (sent out 6 months post partum), and Q5y (sent out when the child was 5 years old); Q2 was a questionnaire about nutrition and was not included in the study. This study was based on data file version 9, released for research in November 2015 and approved by the Regional Committees for Medical and Health Research Ethics, region South-East. The establishment of MoBa and initial data collection were based on a license from the Norwegian Data Protection Agency and approval from the Regional Committees for Medical and Health Research Ethics. The MoBa cohort is currently regulated by the Norwegian Health Registry Act. The Strengthening the Reporting of Observational Studies in Epidemiology (STROBE) reporting guideline was followed.

The Medical Birth Registry of Norway is a national health registry containing information about all births in Norway since 1967, including stillbirths and elective abortions after week 12 of pregnancy.^19^ The NPR contains information about diagnoses and procedures in specialist health care for individual patients from 2008.^20^ Diagnoses are recorded according to the *International Statistical Classification of Diseases and Related Health Problems, Tenth Revision* (*ICD-10*).^21^ The Norwegian Prescription Database contains information about all prescriptions dispensed to individual patients from pharmacies in Norway starting from 2004.^22^ Dispensed medications are coded according to the Anatomical Therapeutic Chemical (ATC) classification system.^23^

### Study Sample

A flowchart of the study sample selection is presented in the Figure. We included live-born singletons whose mother returned the 2 health-related MoBa questionnaires completed during pregnancy (Q1 and Q3). We further restricted the sample to include women who reported migraine or triptan use before or during pregnancy in Q1 or who filled a triptan prescription during the 6 months before pregnancy. Two analytic samples were defined, 1 for each of the specific objectives: an ADHD diagnosis sample and an ADHD symptoms sample. Children of mothers who did not return the 5-year questionnaire (Q5y) or had more than 8 of 12 missing items on the Conners’ Parent Rating Scale (CPRS) were excluded from the ADHD symptoms sample.

**Figure.**
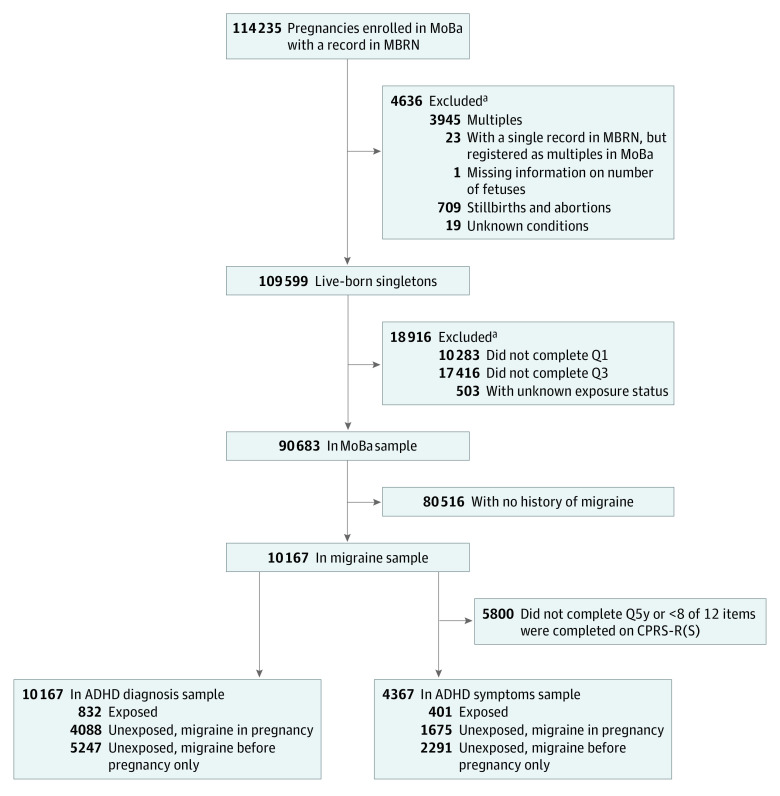
Study Sample Selection ADHD indicates attention-deficit/hyperactivity disorder; CPRS-R(S), Conners’ Parent Rating Scale–Revised, Short Form; MBRN, Medical Birth Registry of Norway; MoBa, Norwegian Mother, Father and Child Cohort Study; and Q, questionnaire. ^a^The conditions of exclusion can overlap.

### Exposure

Women could report use of medications during pregnancy under specific indications in 2 prenatal questionnaires (Q1 and Q3) and 1 postpartum questionnaire (Q4), including migraine (Q1), migraine or headache (Q3), and headache or other pains (Q4). Because triptans are used specifically for migraine, we included triptans reported under any indication in the questionnaires. Use of triptans was coded according to the ATC classification system (code N02CC).^23^ Additional information about exposure is included in the eMethods in the Supplement. We defined 2 triptan-unexposed comparison groups: one group who reported migraine during pregnancy and a second group with migraine before but not during pregnancy. We selected these groups to evaluate the potential association of confounding by indication with our estimates; we expected that the group with migraine only before pregnancy would have less severe migraines than the group with migraine during pregnancy and that any observed association would be stronger for this group if confounding by migraine severity was present.

### Outcomes

#### ADHD Diagnosis

We used both diagnosis and dispensed medication to identify children with ADHD, as has been done in previous studies.^24,25,26^ Age at time of ADHD was defined as the age at the first instance of either a recorded diagnosis of hyperkinetic disorder (*ICD-10* code F90) in the NPR between 2008 and 2015 or a filled prescription of ADHD medication in the Norwegian Prescription Database (ATC code N06BA, except N06BA07, which is not indicated for ADHD) from 2004 to 2016. Children with no event were censored at the end of follow-up. For these children, we did not have the exact age at time of censoring, so their age was estimated by subtracting the birth year from the year that follow-up ended (2016). The term *ADHD diagnosis* used in the rest of the article refers to either a recorded diagnosis or a dispensed medication.

#### ADHD Symptoms

To identify children with problems but who do not necessarily meet the diagnostic criteria for ADHD, we also included ADHD symptoms at 5 years measured by 12 selected items from the Conners’ Parent Rating Scale–Revised, Short Form (CPRS-R[S])^27,28^ in Q5y. The selected items are in the areas of inattention and hyperactivity or impulsivity (eTable 1 in the Supplement), and they have been found to be associated with later diagnosis of ADHD.^29^ Mean scores were calculated for children with at least 8 of 12 items completed and were standardized. Higher *z* scores indicate more ADHD symptoms.

### Covariates

Covariates were identified through use of directed acyclic graphs (eFigure 2 in the Supplement) and categorized as in Table 1. Covariate information came from the Medical Birth Registry of Norway, MoBa, and the Norwegian Prescription Database (eTable 2 in the Supplement). Potential confounders and risk factors for the outcome included maternal age at delivery, parity, marital status, educational level, income, prepregnancy body mass index, folic acid use, smoking during early pregnancy, alcohol use during early pregnancy, pregnancy planning, use of other medications during pregnancy (acetaminophen, nonsteroidal anti-inflammatory drugs, opioids, preventive migraine medications, and psychotropics), maternal and paternal ADHD medication use, child sex assigned at birth, symptoms of anxiety or depression, and satisfaction with life (proxy for migraine severity). Symptoms of anxiety or depression were measured by the 5-item version of the Hopkins Symptoms Checklist^30^ in Q1. Satisfaction with life was measured by the 5 items of the Satisfaction With Life Scale^31^ in Q1. Migraine is associated with disability and reduced quality of life,^32,33^ and a previous study reported reduced life satisfaction among patients with migraine compared with healthy controls.^34^

**Table 1.** Maternal, Paternal, and Child Characteristics by Triptan Exposure Status

Characteristic	ADHD diagnosis sample, No. (%) (n = 10 167)			ADHD symptoms sample, No. (%) (n = 4367)		
	Exposed (n = 832)	Unexposed		Exposed (n = 401)	Unexposed	
		Migraine during pregnancy (n = 4088)	Migraine before pregnancy (n = 5247)		Migraine during pregnancy (n = 1675)	Migraine before pregnancy (n = 2291)
Maternal characteristics						
Age, mean (SD), y	31.0 (4.4)	30.0 (4.7)	30.2 (4.6)	31.3 (4.3)	30.6 (4.4)	30.4 (4.4)
Primiparous	398 (47.8)	1738 (42.5)	2595 (49.5)	205 (51.1)	757 (45.2)	1221 (53.3)
Married or cohabiting	784 (94.2)	3898 (95.4)	4997 (95.2)	379 (94.5)	1606 (95.9)	2202 (96.1)
High educational level^a^	567 (68.1)	2591 (63.4)	3377 (64.4)	300 (74.8)	1218 (72.7)	1654 (72.2)
Missing	7 (0.8)	18 (0.4)	24 (0.5)	5 (1.2)	5 (0.3)	10 (0.4)
Income						
Low	214 (25.7)	1285 (31.4)	1508 (28.7)	85 (21.2)	412 (24.6)	533 (23.3)
Average	487 (58.5)	2188 (53.5)	2985 (56.9)	242 (60.3)	965 (57.6)	1391 (60.7)
High	104 (12.5)	467 (11.4)	577 (11.0)	63 (15.7)	247 (14.7)	310 (13.5)
Missing	27 (3.2)	148 (3.6)	177 (3.4)	11 (2.7)	51 (3.0)	57 (2.5)
Planned pregnancy	630 (75.5)	3206 (78.4)	4150 (79.1)	315 (78.6)	1368 (81.7)	1896 (82.7)
Missing	13 (1.6)	39 (1.0)	57 (1.1)	5 (1.2)	11 (0.7)	15 (0.7)
BMI, mean (SD)	24.6 (4.6)	24.7 (4.9)	24.2 (4.4)	24.4 (4.2)	24.3 (4.6)	24.0 (4.2)
Missing	20 (2.4)	90 (2.2)	130 (2.5)	9 (2.2)	40 (2.4)	33 (1.4)
Folic acid supplement^b^	668 (80.3)	3145 (76.9)	4071 (77.6)	349 (87.0)	1450 (86.6)	1966 (85.8)
Smoking						
Yes	72 (8.7)	384 (9.4)	471 (9.0)	20 (5.0)	103 (6.1)	119 (5.2)
No	654 (78.6)	3103 (75.9)	3926 (74.8)	339 (84.5)	1361 (81.3)	1845 (80.5)
Stopped	97 (11.7)	557 (13.6)	808 (15.4)	41 (10.2)	202 (12.1)	321 (14.0)
Missing	9 (1.1)	44 (1.1)	42 (0.8)	<5	9 (0.5)	6 (0.3)
Alcohol use						
No or minimal	719 (86.4)	3538 (86.5)	4548 (86.8)	347 (86.5)	1482 (88.5)	2031 (88.6)
Yes	20 (2.4)	97 (2.7)	108 (2.3)	12 (3.0)	40 (2.6)	40 (1.9)
Missing	93 (11.2)	453 (11.1)	591 (11.3)	42 (10.5)	153 (9.1)	220 (9.6)
Depression or anxiety symptoms score (SCL-5), mean (SD)^c^	1.3 (0.4)	1.3 (0.4)	1.3 (0.4)	1.3 (0.4)	1.3 (0.4)	1.3 (0.4)
Missing	34 (4.1)	148 (3.6)	166 (3.2)	12 (3.0)	55 (3.3)	60 (2.6)
Gestational hypertension	23 (2.8)	103 (2.5)	129 (2.5)	11 (2.7)	49 (2.9)	56 (2.4)
Preeclampsia						
No	781 (93.9)	3921 (95.9)	5009 (95.5)	378 (94.3)	1617 (96.5)	2187 (95.5)
Mild or unspecified	34 (4.1)	109 (2.7)	159 (3.0)	17 (4.2)	37 (2.2)	68 (3.0)
Severe	17 (2.0)	58 (1.4)	79 (1.5)	6 (1.5)	21 (1.3)	36 (1.6)
Placenta previa	<5	12 (0.3)	16 (0.3)	<5	<5	9 (0.4)
Satisfaction with life score (SWLS), mean (SD)^d^	5.5 (1.1)	5.6 (1.1)	5.6 (1.1)	5.6 (1.0)	5.7 (1.1)	5.7 (1.0)
Missing	21 (2.5)	121 (3.0)	128 (2.4)	8 (2.0)	39 (2.3)	40 (1.7)
ADHD medication use^e^	17 (2.0)	79 (1.9)	89 (1.7)	6 (1.5)	28 (1.7)	25 (1.1)
ADHD symptom score (ASRS), mean (SD)^f^	1.14 (0.60)	1.14 (0.61)	1.12 (0.58)	1.13 (0.61)	1.11 (0.61)	1.09 (0.58)
Missing	305 (36.7)	1794 (35.3)	2215 (42.2)	72 (18.0)	332 (19.8)	434 (18.9)
Other medications^g^						
Acetaminophen	655 (78.8)	3049 (74.6)	3156 (60.1)	317 (79.1)	1260 (75.2)	1384 (60.4)
NSAIDs	179 (21.5)	621 (15.2)	488 (9.3)	92 (22.9)	262 (15.6)	208 (9.1)
Opioids	122 (14.7)	321 (7.9)	166 (3.2)	48 (12.0)	128 (7.6)	64 (2.8)
Preventive migraine medication	13 (1.6)	16 (0.4)	<5	<5	5 (0.3)	<5
Psychotropics	55 (6.6)	152 (3.7)	203 (3.9)	24 (6.0)	52 (3.1)	83 (3.6)
Paternal and child characteristics						
Paternal ADHD medication use^e^	11 (1.3)	43 (1.1)	46 (0.9)	5 (1.3)	15 (0.9)	18 (0.8)
Child sex						
Boy	447 (53.7)	2039 (49.9)	2745 (52.3)	198 (49.4)	809 (48.3)	1184 (51.7)
Girl	385 (46.3)	2049 (50.1)	2502 (47.7)	203 (50.6)	866 (51.7)	1107 (48.3)
Preterm (<37 wk)	41 (4.9)	169 (4.1)	228 (4.3)	18 (4.5)	68 (4.1)	103 (4.5)
Missing	<5	21 (0.5)	21 (0.4)	<5	7 (0.4)	9 (0.4)
Low birth weight (<2500 g)	30 (3.6)	84 (2.1)	158 (3.0)	10 (2.5)	34 (2.0)	72 (3.1)
Missing	<5	6 (0.1)	<5	0	>5	0
Congenital malformations	39 (4.7)	183 (4.5)	241 (4.6)	16 (4.0)	76 (4.5)	103 (4.5)

Abbreviations: ADHD, attention-deficit/hyperactivity disorder; ASRS, Adult ADHD Self-Report Scale; ATC, Anatomical Therapeutic Chemical classification system; BMI, body mass index (calculated as weight in kilograms divided by height in meters squared); NSAIDs, nonsteroidal anti-inflammatory drugs; SCL-5, Hopkins Symptoms Checklist (5-item version); SWLS, Satisfaction With Life Scale.

^a^ College or university education, completed or ongoing.

^b^ Folic acid supplement before pregnancy or during first trimester.

^c^ Mean score per item on SCL-5 (range, 1-4).

^d^ Mean score per item on SWLS (range, 1-7).

^e^ ATC code N06B (Norwegian Prescription Database).

^f^ Mean score per item on ASRS (range, 1-5).

^g^ ATC codes M01A (NSAIDs), N02BE01 (acetaminophen), and N02A (opioids); psychotropic drugs in ATC code groups N05A (antipsychotics), N05BA (benzodiazepines), N05CF (benzodiazepine-like), N06A (antidepressants), and N06BA (stimulants); and preventive migraine medications in groups N06AA (tricyclic antidepressants), N03A (antiepileptics), C07A (β-blockers), C09A (angiotensin-converting enzyme inhibitors), C09C (angiotensin II receptor blockers), and M03AX (botulinum toxin).

### Statistical Analysis

Data were analyzed from May 1 to November 30, 2021. We estimated propensity scores and calculated stabilized inverse probability of treatment weights (IPTWs) to estimate the average treatment effect.^35^ Propensity scores were estimated using logistic regression with the exposure as dependent variable and potential confounders and risk factors for the outcome as independent variables. Including all potential confounders and risk factors for the outcome in the propensity score estimation has demonstrated increased precision without increased bias.^36^ We examined covariate balance after weighting by using standardized differences of 0.1 or more as the cutoff for imbalance.^35^ Nonoverlapping regions of the propensity scores were trimmed.^37^ Stabilized weights were estimated by weighting exposed children by the probability of being exposed divided by their conditional probability of being exposed (ie, the propensity score) and by weighting unexposed children by the probability of being unexposed divided by their conditional probability of being unexposed. Estimates derived from models including IPTWs are referred to as weighted estimates. We used 95% CIs to describe the precision of our estimates, corresponding to an α of .05 for a 2-sided test.

For ADHD diagnosis, Cox proportional hazards regression models with robust SEs were used to estimate crude and weighted hazard ratios (HRs) with 95% CIs. Children were followed up from birth until the first instance of ADHD (diagnosis or dispensed medication) occurring after 3 years of age or censored at the end of follow-up. Year of birth was included in the regression models. Some children died (n = 28) or emigrated (n = 105) during the follow-up period, but we do not know the dates of death or emigration. These children were therefore censored at the time of their most recent appearance in any of the registries or at the end of 2016 if they did not appear in any of the registries. We assessed the proportional hazard assumption by visually examining the hazard curves.

For ADHD symptoms, we estimated censoring weights in addition to treatment weights to account for loss to follow-up because only 43.0% of mothers (4367 of 10 167) returned Q5y and completed the CPRS-R(S). The probability of being uncensored was estimated using logistic regression. Only variables with complete information were included in the censoring model. Stabilized inverse probability of censoring weights (IPCWs) were estimated and multiplied with IPTWs, resulting in a combined weight in which participants in the 5-year sample were upweighted to represent participants who were lost to follow-up.^38^ Generalized linear models with robust SEs were used to estimate crude and weighted mean differences in CPRS-R(S) *z* scores with 95% CIs.

Multiple imputation by chained equations^39^ with 30 imputations was used to impute missing values in covariates, under the assumption of missing at random. Additional information about the imputation procedure can be found in the eMethods in the Supplement. Stata, version 16.1 (StataCorp LLC)^40^ was used in all analyses.

We conducted several sensitivity analyses. Because not all children with ADHD are treated with medication, we repeated the analyses using only the diagnosis from the NPR to assess ADHD among children born in or after 2008. To examine whether children with missing items on the CPRS-R(S) may have more symptoms of ADHD, we restricted the analysis of ADHD symptoms to children with no missing items. To assess the robustness of our models, we included different model specifications of the IPTWs, including models with prematurity and low birth weight (these were considered mediators in the main analysis and were therefore not included), models without paternal and child factors, and models with maternal ADHD symptoms, measured by the Adult ADHD Self-Report Scale^41^ (not included in the main analysis owing to the high amount of missing information). We also compared results based on trimmed and untrimmed weights. To examine potential exposure misclassification, we applied probabilistic bias analysis with different scenarios for sensitivity and specificity (additional information can be found in eMethods in the Supplement).^42^ Finally, we did a complete-case analysis as a comparison with the multiple imputation analysis.

## Results

Mothers were classified as having migraine before or during pregnancy in 11.2% of the pregnancies (10 167 of 90 683). Of the women with migraine during pregnancy, 86.8% (4188 of 4823) also reported migraine before pregnancy. The ADHD diagnosis sample consisted of 10 167 children (5231 boys [51.5%]) born to 8412 mothers (mean [SD] age, 30.2 [4.6] years) with migraine before or during pregnancy. The ADHD symptoms sample consisted of 4367 children (2191 boys [50.2%]) born to 3855 mothers (mean [SD] maternal age, 30.6 [4.4] years) with migraine before or during pregnancy. In the ADHD diagnosis sample, 832 children (8.2%) were exposed to triptans during pregnancy. In the ADHD symptoms sample, 401 children (9.2%) were exposed to triptans during pregnancy. Characteristics of the samples are presented in Table 1. Exposed women were slightly older and more often highly educated. They were also less likely to smoke during pregnancy and more often had a planned pregnancy. After weighting, all covariates were balanced between exposed and unexposed children (standardized mean differences <0.1). Weights had a mean of 1 and an acceptable range (eTable 3 in the Supplement). A total of 21.0% of the sample (2130 of 10 167) had missing values in any of the potential confounders or risk factors.

### ADHD Diagnosis

The mean (SD) age at time of diagnosis was 8.6 (2.1) years (range, 3.6-16.0 years), and children were followed up for a mean (SD) of 10.6 (2.2) years (range, 0.7-16.0 years). In total, 377 of 10 167 (3.7%) children received a diagnosis of ADHD. Prenatal exposure to triptans was not associated with an increased risk of ADHD compared with unexposed children whose mothers had migraine during pregnancy (weighted HR, 1.16; 95% CI, 0.78-1.74) and compared with unexposed children whose mothers had migraine only before pregnancy (weighted HR, 1.28; 95% CI, 0.84-1.94) (Table 2). The hazards for exposed and unexposed children seemed proportional for most of the follow-up time, except for some deviations from proportionality at the beginning and end of follow-up, where there were few ADHD cases (eFigure 3 in the Supplement). At the end of follow-up, there were also fewer children at risk because of how we estimated the age of children with no event.

**Table 2.** Associations Between Prenatal Triptan Exposure and Childhood ADHD Diagnosis

Characteristic	Total No.	ADHD cases, No.	IR per 1000 person-years (95% CI)	Hazard ratio (95% CI)^a^	
				Crude	Weighted^b^
Triptan use during pregnancy	832	33	3.8 (2.7-5.3)	1.07 (0.74-1.56)	1.16 (0.78-1.74)
Migraine during pregnancy	4088	160	3.7 (3.1-4.3)	1 [Reference]	1 [Reference]
Triptan use during pregnancy	832	33	3.8 (2.7-5.3)	1.15 (0.79-1.67)	1.28 (0.84-1.94)
Migraine prior to pregnancy	5247	184	3.3 (2.9-3.9)	1 [Reference]	1 [Reference]

Abbreviations: ADHD, attention-deficit/hyperactivity disorder; IR, incidence rate.

^a^ Estimated using trimmed weights (triptan use during pregnancy vs migraine during pregnancy [6 excluded]; triptan use during pregnancy vs migraine before pregnancy [53 excluded]).

^b^ Weighted estimates were adjusted for maternal age, parity, marital status, educational level, income, body mass index, folic acid use, smoking habits, alcohol use, pregnancy planning, use of other medications during pregnancy, maternal and paternal ADHD medication use, symptoms of anxiety or depression, satisfaction with life, and child’s sex.

### ADHD Symptoms

The mean (SD) CPRS-R(S) score was 1.39 (0.4) (range, 1-4), and 5.5% of the children (241 of 4367) had a *z* score 2 or more SDs from the mean, which is considered to indicate clinically relevant problems.^43^ Children with ADHD had a mean (SD) CPRS-R(S) score at 5 years of 2.0 (0.8) (eTable 4 in the Supplement), and 39.1% (45 of 115) had *z* scores above the clinical cutoff. There were no differences in mean *z* scores between triptan-exposed children and triptan-unexposed children whose mothers had migraines during pregnancy (weighted mean difference, −0.11; 95% CI, −0.25 to 0.04) or between triptan-exposed children and triptan-unexposed children whose mothers had migraines only before pregnancy (weighted mean difference, −0.09; 95% CI, −0.24 to 0.07) (Table 3).

**Table 3.** Associations Between Prenatal Triptan Exposure and Childhood ADHD Symptoms at 5 Years

Characteristic	No.	CPRS-R(S) score, mean (SD)	CPRS-R(S) *z* score, mean (SD)	Difference in *z* scores, mean (95% CI)^a^	
				Crude	Weighted^b^
Triptan use during pregnancy	401	1.37 (0.41)	0.01 (1.06)	−0.05 (−0.17 to 0.07)	−0.11 (−0.25 to 0.04)
Migraine during pregnancy	1675	1.39 (0.42)	0.07 (1.11)	0 [Reference]	0 [Reference]
Triptan use during pregnancy	401	1.37 (0.41)	0.01 (1.06)	−0.08 (−0.19 to 0.03)	−0.09 (−0.24 to 0.07)
Migraine prior to pregnancy	2291	1.40 (0.41)	0.08 (1.06)	0 [Reference]	0 [Reference]

Abbreviations: ADHD, attention-deficit/hyperactivity disorder; CPRS-R(S), Conners’ Parents Rating Scale–Revised, Short Form.

^a^ Mean differences were estimated using trimmed weights (triptan use during pregnancy vs migraine during pregnancy [8 excluded]; triptan use during pregnancy vs migraine before pregnancy [38 excluded]).

^b^ Weighted estimates were adjusted for maternal age, parity, marital status, educational level, income, body mass index, folic acid use, smoking habits, alcohol use, pregnancy planning, use of other medications during pregnancy, maternal and paternal ADHD medication use, symptoms of anxiety or depression, satisfaction with life, and child’s sex.

### Sensitivity Analyses

Probabilistic bias analysis of nondifferential exposure misclassification showed that specificity would have to be unrealistically low to change our conclusion. In realistic scenarios, bias due to nondifferential exposure misclassification could have biased our results by 2% toward the null (eTable 5 in the Supplement). When restricting the analyses to children with data available from the NPR, we found a (1) lower incidence of ADHD and (2) crude and weighted HRs within the 95% CIs of the estimates from the main results (eTable 6 in the Supplement); however, there were only 2 exposed cases and wide 95% CIs. Using models with different specifications of IPTWs resulted in similar weight characteristics (eTable 3 in the Supplement) and similar estimates (eFigure 4 and eFigure 5 in the Supplement) as the main models. Results from other sensitivity analyses were generally comparable with the main analyses (eResults, eTable 7, and eFigures 6-9 in the Supplement)

## Discussion

In this study of pregnant women with migraine and their children, we found no associations between prenatal exposure to triptans and ADHD among offspring, both when examining ADHD diagnosis and maternal-reported ADHD symptoms at 5 years. These results are reassuring for women in need of triptans during pregnancy.

With a mean follow-up time of 10.6 years, we found a prevalence of ADHD that was similar to the prevalence reported for children aged 12 years in Norway (3.8%).^44^ The risk of ADHD after prenatal triptan exposure has not been examined previously, to our knowledge. One study assessed the risk of autism spectrum disorders, finding no association with prenatal triptan exposure.^45^ Other studies investigated associations between prenatal triptan exposure and parent-reported neurodevelopmental outcomes in children up to 5 years.^5,11,12,13^ Studies of young children found associations of prenatal triptan exposure with increased risk of externalizing behavior problems,^11^ which includes a measure of hyperactivity, and with increased activity and emotionality.^12^ These associations were not seen in 5-year-old children,^13^ which may suggest that early externalizing-type behavior problems after prenatal triptan exposure are not persistent. This is in line with the findings in this study. However, another possible explanation could be bias due to confounding by migraine severity in studies of younger children, which could have led to overestimation of the risk of externalizing-type behavior problems associated with prenatal triptan exposure in these studies. One study used propensity score calibration to adjust for migraine severity among mothers of 3-year-old children and found no increased risk of communication problems, motor problems, or temperament differences; adjustment for migraine severity reduced the effect estimates by 2% to 50% in this study.^5^ A second explanation for the different findings for 3-year-old and 5-year-old children could be more pronounced bias from loss to follow-up in 5-year-old children, particularly if children with neurodevelopmental problems are more likely to be lost to follow-up from the study. However, in the present study, we found similar results for ADHD symptoms and ADHD diagnosis, for which there was no loss to follow-up. Most of the studies of prenatal triptan exposure and child neurodevelopment have been conducted in the MoBa cohort in Norway, where access to health care is free and the educational level is high,^46^ and future studies should include cohorts from other countries with different health care and socioeconomic structures.

### Strengths and Limitations

This study has some strengths, including the prospective design and long follow-up time in MoBa, with access to detailed information about potential confounders of the association between prenatal triptan exposure and childhood ADHD. In addition, we were able to assess both ADHD diagnosis and symptoms. Including the symptom perspective is important because an additional 5% of children beyond those with a diagnosis of ADHD did not meet the full diagnostic criteria but still had symptoms of ADHD according to a review of neurodevelopment outcome measures.^15^ Moreover, we used modern methods to account for potential biases from loss to follow-up, confounding, and missing data.

This study also has some limitations. First, misclassification of exposure may have affected the results. We expect any misclassification to be nondifferential with bias toward the null, which could have led to not detecting an actual increased risk of ADHD. If a woman reported use of multiple migraine medications and indicated use both before and during pregnancy in Q1, we assumed that she was exposed to all the reported migraine medications before and during pregnancy, even though that might not have been the case. In Q4, triptan exposure was reported retrospectively 6 months after birth, and there was no specific migraine indication mentioned in this questionnaire. Reporting of late pregnancy exposure could therefore be subject to poor recall. However, the probabilistic bias analysis showed that the specificity would have had to be very low to change our conclusion. We did not have information about dosage, and dose-dependent effects cannot be ruled out. Another limitation is that migraine was self-reported. However, according to a previous study, self-reported migraine was confirmed in 82% of pregnant women.^47^ Potential misclassification also applies to the outcomes. Attention-deficit/hyperactivity disorder symptoms were reported by parents and might be associated with maternal migraine and childhood ADHD. However, the mean symptom scores corresponded well with ADHD diagnosis, given that they were measured at the mean age of 5 years and 9 years, respectively, and we observed similar results. The validity of ADHD diagnosis in the NPR has not been established, but a study from Denmark found a positive predictive value of 0.87 for ADHD diagnoses in children registered in the Danish Psychiatric Central Research Registry.^48^

Second, there could be bias from self-selection and loss to follow-up in MoBa. The participation rate was 41%, and participants were less likely to be young mothers and more likely to be married or cohabiting, have a high educational level, and have a healthier lifestyle during pregnancy compared with the general population.^49^ This finding affects the generalizability of our results and could affect the association between prenatal exposures and childhood ADHD, according to a previous study.^50^ Even though we used IPCW to address loss to follow-up as recommended, there could be additional factors associated with dropout not included in the censoring model, such as migraine and ADHD. In addition, we were not able to include educational level as a factor when the IPCWs were combined with IPTWs estimated from imputed data, which is a limitation, because educational level has been found to be an important factor associated with participation in MoBa.^50^

Third, although we took several measures to address bias from confounding by indication, this could have affected our results. Migraine severity is likely to be an important confounder; women using triptans during pregnancy are likely to have more severe migraines than women not using triptans, and migraines are heritable and associated with ADHD in children.^51,52^ A recent study using quantitative genetic modeling concluded that the association between migraines and ADHD was almost completely explained by genetic factors.^53^ We included 2 comparison groups with expected different migraine severity, finding no systematic differences between the groups. Moreover, we tried to account for migraine severity by using satisfaction with life as a proxy. However, this measure is not specific to migraines and may not reflect differences in migraine severity, although the unexposed groups had slightly higher mean scores than the exposed group.

Fourth, we had too few exposed children to be able to investigate triptan exposure by trimester or to examine specific triptans. For the same reason, we cannot rule out small effects.

## Conclusions

This cohort study found no association between prenatal triptan exposure and ADHD diagnosis or ADHD symptoms at 5 years of age. This study adds to the growing literature on the safety of triptan use during pregnancy and expands it to an important neurobehavioral outcome.

## Notes

Supplement.
eMethods.

eResults.
**eTable 1.** Items from the Conners’ Parent Rating Scale-Revised, Short Form Included in Q5y **eTable 2.** Overview of Covariates **eTable 3.** Characteristics of Weights Using Different Model Specifications **eTable 4.** CPRS Score According to ADHD Diagnosis **eTable 5.** Probabilistic Bias Analysis of Non-Differential Misclassification of Exposure in the Weighted ADHD Diagnosis Analysis, Using Trapezoidal Distributions for Sensitivities and Specificities According to Different Scenarios **eTable 6.** Associations Between Prenatal Triptan Exposure and Child ADHD Diagnosis Using Only Data From NPR to Assess ADHD Diagnosis and Restricting to Children Born in or After 2008 **eTable 7.** Associations Between Prenatal Triptan Exposure and Child ADHD Symptoms, Restricted to Children With No Missing Items on the CPRS **eFigure 1.** Measures of ADHD According to Data Source and Time Coverage **eFigure 2.** Proposed Causal Model for the Association Between Triptan Use During Pregnancy and Child ADHD **eFigure 3.** Weighted Kaplan-Meier Failure Curves Showing the Proportion With ADHD Diagnosis Among Exposed and Unexposed **eFigure 4.** Comparing the Association Between Prenatal Triptan Exposure and Child ADHD Diagnosis Based on Different Specifications of Inverse Probability of Treatment Weights **eFigure 5.** Comparing the Association Between Prenatal Triptan Exposure and Child ADHD Symptoms Based on Different Specifications of Inverse Probability of Treatment Weights **eFigure 6.** Comparing the Association Between Prenatal Triptan Exposure and Child ADHD Diagnosis Using Trimmed vs Untrimmed Weights **eFigure 7.** Comparing the Association Between Prenatal Triptan Exposure and Child ADHD Symptoms Using Trimmed vs Untrimmed Weights **eFigure 8.** Comparing the Association Between Prenatal Triptan Exposure and Child ADHD Diagnosis Based on Imputed Data vs Complete Cases **eFigure 9.** Comparing the Association Between Prenatal Triptan Exposure and Child ADHD Symptoms Based on Imputed Data vs Complete Cases
eReferences.
