# Supplementary material for: Association of Maternal Use of Triptans During Pregnancy With Risk of Attention-Deficit/Hyperactivity Disorder in Offspring
Source: JAMA Netw Open. 2022 Jun 3;5(6):e2215333. doi: 10.1001/jamanetworkopen.2022.15333 (PMC9166221; doi:10.1001/jamanetworkopen.2022.15333)
Supplement: Supplement. — eMethods. eResults. eTable 1. Items from the Conners’ Parent Rating Scale-Revised, Short Form Included in Q5y eTable 2. Overview of Covariates eTable 3. Characteristics of Weights Using Different Model Specifications eTable 4. CPRS Score According to ADHD Diagnosis eTable 5. Probabilistic Bias Analysis of Non-Differential Misclassification of Exposure in the Weighted ADHD Diagnosis Analysis, Using Trapezoidal Distributions for Sensitivities and Specificities According to Different Scenarios eTable 6. Associations Between Prenatal Triptan Exposure and Child ADHD Diagnosis Using Only Data From NPR to Assess ADHD Diagnosis and Restricting to Children Born in or After 2008 eTable 7. Associations Between Prenatal Triptan Exposure and Child ADHD Symptoms, Restricted to Children With No Missing Items on the CPRS eFigure 1. Measures of ADHD According to Data Source and Time Coverage eFigure 2. Proposed Causal Model for the Association Between Triptan Use During Pregnancy and Child ADHD eFigure 3. Weighted Kaplan-Meier Failure Curves Showing the Proportion With ADHD Diagnosis Among Exposed and Unexposed eFigure 4. Comparing the Association Between Prenatal Triptan Exposure and Child ADHD Diagnosis Based on Different Specifications of Inverse Probability of Treatment Weights eFigure 5. Comparing the Association Between Prenatal Triptan Exposure and Child ADHD Symptoms Based on Different Specifications of Inverse Probability of Treatment Weights eFigure 6. Comparing the Association Between Prenatal Triptan Exposure and Child ADHD Diagnosis Using Trimmed vs Untrimmed Weights eFigure 7. Comparing the Association Between Prenatal Triptan Exposure and Child ADHD Symptoms Using Trimmed vs Untrimmed Weights eFigure 8. Comparing the Association Between Prenatal Triptan Exposure and Child ADHD Diagnosis Based on Imputed Data vs Complete Cases eFigure 9. Comparing the Association Between Prenatal Triptan Exposure and Child ADHD Symptoms Based on Imputed Data vs Complete Cases eReferenc [file jamanetwopen-e2215333-s001.pdf]

Supplementary Online Content

Harris GM, Wood M, Ystrom E, Nordeng H. Association of maternal use of triptans during pregnancy with risk of attention-deficit/hyperactivity disorder in offspring. *JAMA Netw Open.* 2022;5(6):e2215333. doi:10.1001/jamanetworkopen.2022.15333

eMethods.
eResults.
eTable 1. Items from the Conners' Parent Rating Scale-Revised, Short Form Included in Q5y
eTable 2. Overview of Covariates
eTable 3. Characteristics of Weights Using Different Model Specifications
eTable 4. CPRS Score According to ADHD Diagnosis
eTable 5. Probabilistic Bias Analysis of Non-Differential Misclassification of Exposure in the Weighted ADHD Diagnosis Analysis, Using Trapezoidal Distributions for Sensitivities and Specificities According to Different Scenarios
eTable 6. Associations Between Prenatal Triptan Exposure and Child ADHD Diagnosis Using Only Data From NPR to Assess ADHD Diagnosis and Restricting to Children Born in or After 2008
eTable 7. Associations Between Prenatal Triptan Exposure and Child ADHD Symptoms, Restricted to Children With No Missing Items on the CPRS
eFigure 1. Measures of ADHD According to Data Source and Time Coverage
eFigure 2. Proposed Causal Model for the Association Between Triptan Use During Pregnancy and Child ADHD
eFigure 3. Weighted Kaplan-Meier Failure Curves Showing the Proportion With ADHD Diagnosis Among Exposed and Unexposed
eFigure 4. Comparing the Association Between Prenatal Triptan Exposure and Child ADHD Diagnosis Based on Different Specifications of Inverse Probability of Treatment Weights
eFigure 5. Comparing the Association Between Prenatal Triptan Exposure and Child ADHD Symptoms Based on Different Specifications of Inverse Probability of Treatment Weights
eFigure 6. Comparing the Association Between Prenatal Triptan Exposure and Child ADHD Diagnosis Using Trimmed vs Untrimmed Weights
eFigure 7. Comparing the Association Between Prenatal Triptan Exposure and Child ADHD Symptoms Using Trimmed vs Untrimmed Weights
eFigure 8. Comparing the Association Between Prenatal Triptan Exposure and Child ADHD Diagnosis Based on Imputed Data vs Complete Cases
eFigure 9. Comparing the Association Between Prenatal Triptan Exposure and Child ADHD Symptoms Based on Imputed Data vs Complete Cases
eReferences.

This supplementary material has been provided by the authors to give readers additional information about their work.

eMethods.

Additional information about exposure

Women could report use of medications during pregnancy in two prenatal (Q1 and Q3) and one post-partum questionnaire (Q4). In each questionnaire, women were provided with a list of different illnesses and health problems, and asked to indicate if they had the problem/illness. On the same line, they could write the name of any medication used, and indicate when it was used. Q1 covered the period six months before pregnancy and gestational week (GW) 0-4, 5-8, 9-12, and 13+. Q3 covered GW 13-16, 17-20, 21-24, 25-28, and 29+. Q4 covered the last part of pregnancy. The questionnaires are available at the Norwegian Institute of Public Health webpage: https://www.fhi.no/en/studies/moba/for-forskere-artikler/questionnaires-from-moba/.[1]

Additonal information about statistical analyses

Multiple imputation by chained equations was used to impute missing data in covariates, with 30 imputations.[2,3] Imputation models were fitted separately for the ADHD diagnosis sample and ADHD symptoms sample, and included all covariates that were included in the weights, exposure variables and outcome variables. Treatment weights were estimated and outcome models fit in each imputed dataset, then estimates were combined.[4] In the imputation model for ADHD diagnosis we included the hazard function, as recommended.[5]

In the main analysis, non-overlapping regions of the propensity scores of exposed and unexposed were trimmed by excluding individuals with a propensity score outside of the common range, to avoid extrapolation.[6] The number of individuals that were trimmed differed for each imputed dataset, but was fewer than 1% of the total ADHD diagnosis sample and fewer than 2% of the ADHD symptoms sample.

For the probabilistic bias analysis, we used trapezoidal distributions for sensitivity and specificity with 20 000 simulations. Sensitivity and specificity was assumed the same for cases and non-cases (non-differential misclassification). We were not able to identify studies reporting sensitivity and specificity for self-reported triptan use compared to interview or medication diary as gold standard. One study examined the validity of prescribed pain medication, mostly NSAIDs, and found sensitivity between 0.29-0.34 and specificity between 0.98-0.99.[7] Recall time was longer in that study compared to this study, and we expect triptans to have higher sensitivity than NSAIDs because the indication for use is very specific. Another study reported sensitivity of 0.63 for occasional or short-term use of analgesics or antipyretics.[8] Based on this, we defined several scenarios for the parameters of sensitivity and specificity (e.g. optimistic, realistic and pessimistic).

© 2022 Harris GM et al. *JAMA Network Open.*

eResults.

Additonal results from sensitivity analyses

When we restricted the ADHD symptoms analysis to children with no missing items on the Conner's Parent Rating Scale (CPRS), results were similar to the main analysis (eTable 8).

Estimates based on trimmed and untrimmed weights were similar (eFigure 6 and 7).

Results from complete case analysis showed similarities and differences to the main analysis (eFigure 8 and 9). For ADHD symptoms, crude estimates were similar, but for the weighted estimates triptan exposed children had significantly lower scores than children whose mothers had migraine during pregnancy in the complete case analysis. However, the difference was small and not likely to be of clinical relevance.

© 2022 Harris GM et al. *JAMA Network Open.*

eTable 1. Items from the Conners' Parent Rating Scale-Revised, Short Form (CPRS-R(S))[9,10] Included in Q5y

Item	Question<br>*How much of a problem has this been the last month?*	Response	Chronbach's α
1	Inattentive, easily distracted	1 = Not true at all<br>2 = Just a little true<br>3 = Pretty much true<br>4 = Very much true	0.88
2	Short attention span		
3	Fidgets with hands or feet, squirms in seat		
4	Messy or disorganised at home or in the kindergarten		
5	Only attends if it is something he/she is very interested in		
6	Distractibility or attention span a problem		
7	Avoids, expresses reluctance about, or has difficulties engaging in tasks that require sustained mental effort (such as activities in kindergarten or helping out at home)		
8	Gets distracted when given instructions to do something		
9	Has trouble concentrating in kindergarten		
10	Leaves seat in kindergarten or in other situations in which remaining seated is expected		
11	Does not follow through on instructions and fails to finish tasks such as putting away shoes/tidying toys (not due to oppositional behaviour or failure to understand instructions)		
12	Easily frustrated in efforts		

© 2022 Harris GM et al. *JAMA Network Open.*

eTable 2. Overview of Covariates

Variable	Source	Categorization/comment
**Potential confounders:**		
Maternal age	MBRN	Continuous
Marital status	MBRN	Married/cohabiting or other
Parity	MBRN	Primiparous or multiparous
Education	MoBa Q1	College/university or other (completed or ongoing)
Income	MoBa Q1	Average, low, or high
Pre-pregnancy BMI	MoBa Q1	Continuous
Folate intake	MoBa Q1	Yes (before/during pregnancy) or no
Smoking in early pregnancy	MoBa Q1	Yes, no, or stopped
Alcohol use in early pregnancy	MoBa Q1	Yes or no/minimal
Planned pregnancy	MoBa Q1	Yes or no
Symptoms of anxiety and depression (SCL-5)	MoBa Q1	Continuous (mean score)
Maternal ADHD medication	NorPD	Yes or no
Maternal ADHD symptoms (ASRS)	MoBa Q6	Continuous (mean score)
Satisfaction with life (SWLS)	MoBa Q1	Continuous (mean score). Used as proxy for migraine severity.
Use of other medications during pregnancy<br>- Paracetamol<br>- NSAIDs<br>- Opioids<br>- Preventive migraine medications<br>- Psychotropic drugs	MoBa Q1, Q3, Q4	Yes or no
**Risk factors:**		
Paternal ADHD medication	NorPD	Yes or no
Child sex	MBRN	Boy or girl
**Possible mediators:**		
Gestational hypertension	MBRN	Yes or no
Preeclampsia	MBRN	Mild, severe, or none
Placenta previa	MBRN	Yes or no
Birth weight	MBRN	<2500 g or ≥2500 g
Prematurity	MBRN	Yes (<37 weeks) or no (≥37 weeks)
Congenital malformations	MBRN	Yes or no

BMI, body mass index; MBRN, The Medical Birth Registry of Norway; NorPD, Norwegian Prescription Database; SCL-5, Symptoms Check List; ASRS, Adult ADHD Self-Report Scale; SWLS, Satisfaction With Life Scale

© 2022 Harris GM et al. *JAMA Network Open.*

eTable 3. Characteristics of Weights Using Different Model Specifications

**Model specification**	**Estimated IPTW (ADHD diagnosis)**		**Estimated IPTW*IPCW (ADHD symptoms)**	
	**Mean (SD)**	**Min-max**	**Mean (SD)**	**Min-max**
Triptan exposed vs. migraine in pregnancy				
Main model: All potential confounders and risk factors for the outcome	1.0 (0.18)	0.25-4.0	1.0 (0.38)	0.25-5.1
Alternative model 1: Including maternal ADHD symptoms	1.0 (0.18)	0.25-4.2	1.0 (0.38)	0.25-5.3
Alternative model 2: Not including paternal and child factors	1.0 (0.18)	0.26-3.7	1.0 (0.37)	0.25-5.3
Alternative model 3: Including prematurity and low birth weight (considering them as risk factors rather than mediators)	1.0 (0.18)	0.25-3.5	1.0 (0.37)	0.25-5.2
Triptan exposed vs. migraine before pregnancy				
Main model: All potential confounders and risk factors for the outcome	1.0 (0.25)	0.16-6.3	1.0 (0.42)	0.19-9.0
Alternative model 1: Including maternal ADHD symptoms	1.0 (0.26)	0.15-7.0	1.0 (0.40)	0.19-8.6
Alternative model 2: Not including paternal and child factors	1.0 (0.26)	0.16-6.5	1.0 (0.41)	0.19-9.0
Alternative model 3: Including prematurity and low birth weight (considering them as risk factors rather than mediators)	1.0 (0.25)	0.16-6.6	1.0 (0.43)	0.18-9.1

IPTW, Inverse Probability of Treatment Weight, IPCW; Inverse Probability of Censoring Weight

© 2022 Harris GM et al. *JAMA Network Open.*

eTable 4. CPRS Score According to ADHD Diagnosis

ADHD diagnosis	No	Mean score (SD)	Mean z-score (SD)
Yes	115	2.01 (0.75)	1.69 (1.94)
No	4252	1.38 (0.39)	0.03 (1.01)

CPRS; Conners Parent Rating Scale; SD, standard deviation; CI, confidence interval

eTable 5. Probabilistic Bias Analysis of Non-Differential Misclassification of Exposure in the Weighted ADHD Diagnosis Analysis, Using Trapezoidal Distributions for Sensitivities and Specificities According to Different Scenarios

	**Sensitivity**				**Specificity**				**Corrected estimate[a], RR (95% CI)**	**Bias[b], %**
	**Min**	**M1**	**M2**	**Max**	**Min**	**M1**	**M2**	**Max**		
Optimistic scenario 1	0.70	0.75	0.85	1.0	0.95	0.98	0.99	1.0	1.04 (0.70, 1.53)	1.0
Optimistic scenario 2	0.70	0.75	0.80	0.85	0.95	0.98	0.99	1.0	1.04 (0.70, 1.53)	1.0
Realistic scenario 1	0.30	0.60	0.70	0.90	0.90	0.95	0.98	1.0	1.05 (0.71, 1.54)	1.9
Realistic scenario 2	0.50	0.60	0.70	0.80	0.90	0.92	0.95	0.97	1.05 (0.71, 1.55)	1.9
Pessimistic scenario 1	0.15	0.20	0.30	0.50	0.70	0.80	0.90	1.0	1.20 (0.77, 3.40)	14.2
Pessimistic scenario 2	0.30	0.40	0.50	0.60	0.70	0.80	0.85	0.90	1.31 (0.82, 5.95)	21.4
Pessimistic scenario 3	0.30	0.40	0.50	0.60	0.60	0.70	0.80	0.85	2.09 (1.10, 17.9)	50.7

Conventional estimate = 1.03 (0.69-1.51). Migraine during pregnancy was used as comparison group.
M, mode; RR, rate ratio; CI, confidence interval. [a] Median bias-adjusted estimate with 95% simulation limits [b] Calculated as ((corrected-conventional)/corrected)*100

© 2022 Harris GM et al. *JAMA Network Open.*

eTable 6. Associations Between Prenatal Triptan Exposure and Child ADHD Diagnosis Using Only Data From NPR to Assess ADHD Diagnosis and Restricting to Children Born in or After 2008

	**No**	**ADHD cases, No**	**IR per 1000 person-years (95% CI)**	**Crude HR (95% CI)**	**Weighted HR (95% CI)**
Triptans in pregnancy	132	2	1.9 (0.5, 7.8)	1.67 (0.34, 8.22)	1.45 (0.30, 7.22)
Migraine in pregnancy	657	6	1.2 (0.5, 2.6)	1 (reference)	1 (reference)
Triptans in pregnancy	132	2	1.9 (0.5, 7.8)	-	-
Migraine prior to pregnancy	955	7	0.9 (0.4, 2.0)	-	-

IR, incidence rate; HR, hazard ratio; CI, confidence interval

© 2022 Harris GM et al. *JAMA Network Open.*

eTable 7. Associations Between Prenatal Triptan Exposure and Child ADHD Symptoms, Restricted to Children With no Missing Items on the CPRS

	**No**	**Mean CPRS score (SD)**	**Mean CPRS z-score (SD)**	**Crude mean difference in z-scores[a] (95% CI)**	**Weighted mean difference in z-scores[a] (95% CI)**
Triptans in pregnancy	388	1.37 (0.39)	0.00 (1.03)	-0.05 (-0.17, 0.07)	-0.11 (-0.25, 0.03
Migraine in pregnancy	1609	1.39 (0.42)	0.05 (1.11)	0 (reference)	0 (reference)
Triptans in pregnancy	388	1.37 (1.39)	0.00 (1.03)	-0.07 (-0.18, 0.05)	-0.07 (0.23, 0.09)
Migraine prior to pregnancy	2197	1.39 (0.40)	0.07 (1.05)	0 (reference)	0 (reference)

CPRS; Conners Parent Rating Scale; SD, standard deviation; CI, confidence interval

© 2022 Harris GM et al. *JAMA Network Open.*

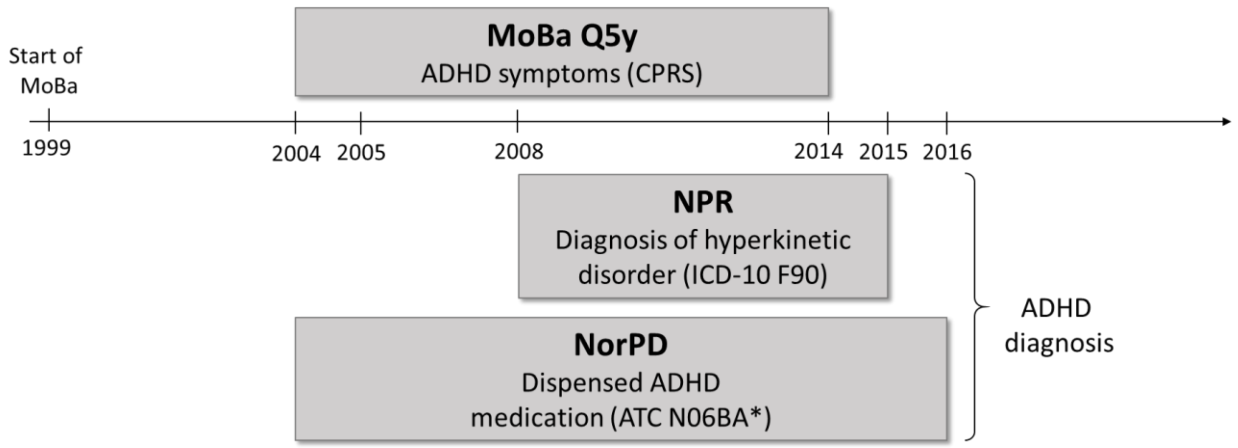

eFigure 1. Measures of ADHD According to Data Source and Time Coverage
*Not including modafinil

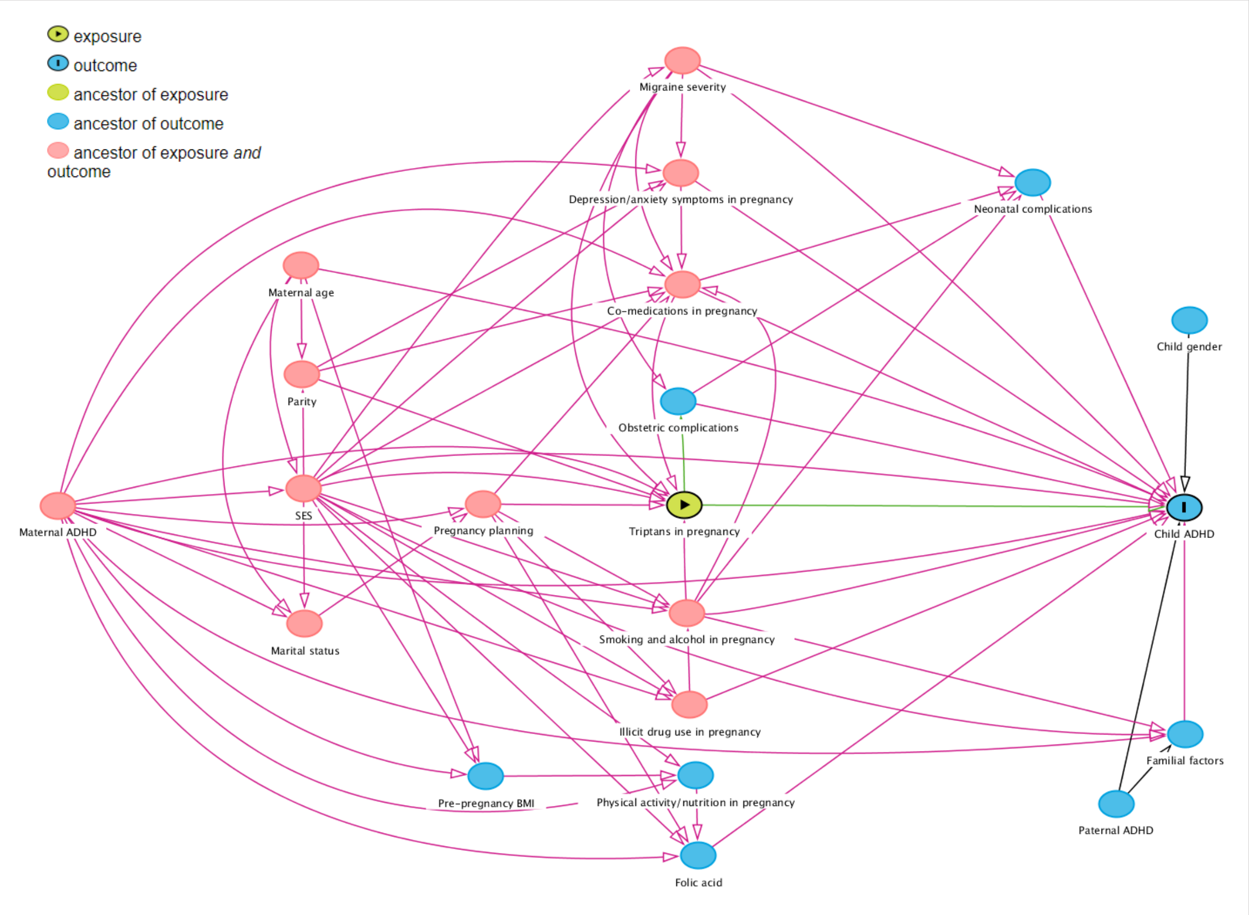

eFigure 2. Proposed Causal Model for the Association Between Triptan Use During Pregnancy and Child ADHD

Socioeconomic status (SES) includes education and income. Neonatal complications include congenital malformations, preterm birth and low birthweight. Obstetric complications include gestational hypertension, preeclampsia and placenta previa. Biasing paths in pink; causal paths in green. The figure was made using www.dagitty.net.

© 2022 Harris GM et al. *JAMA Network Open.*

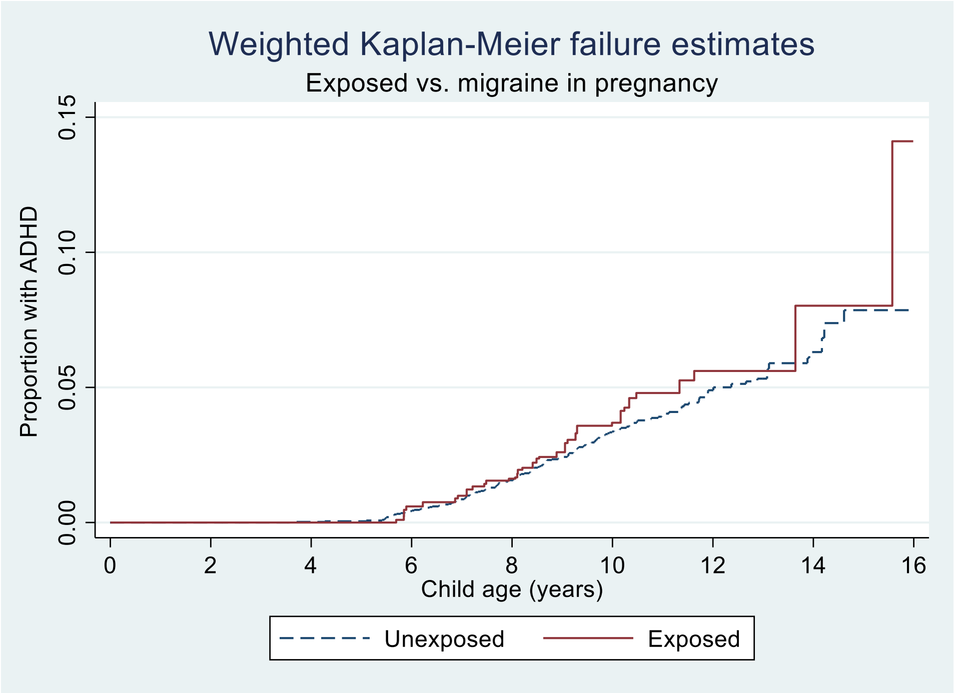

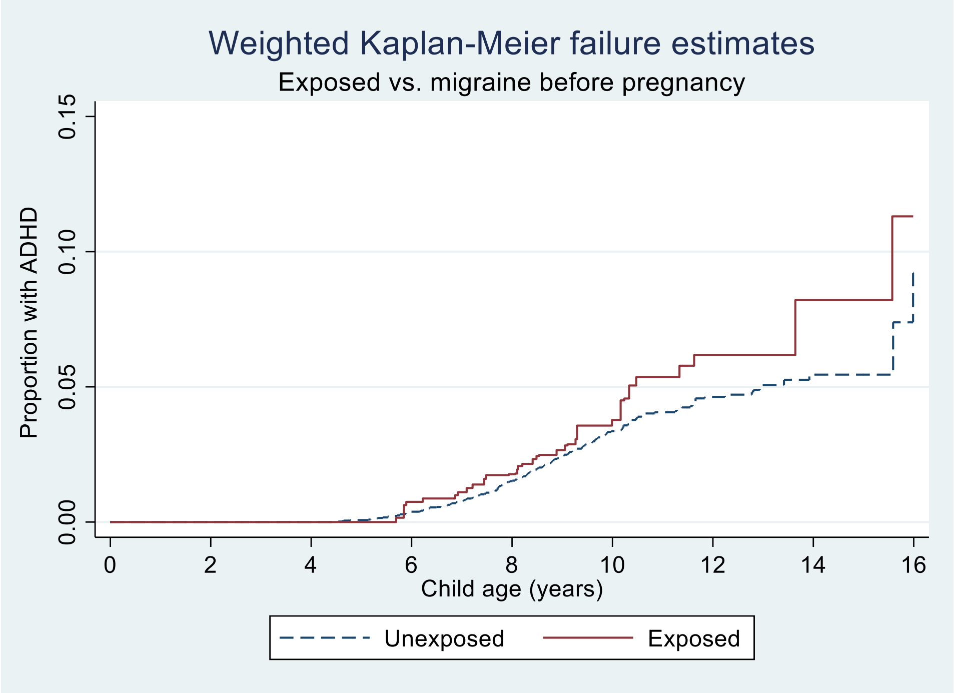

eFigure 3. Weighted Kaplan-Meier Failure Curves Showing the Proportion With ADHD Diagnosis Among Exposed and Unexposed

© 2022 Harris GM et al. *JAMA Network Open.*

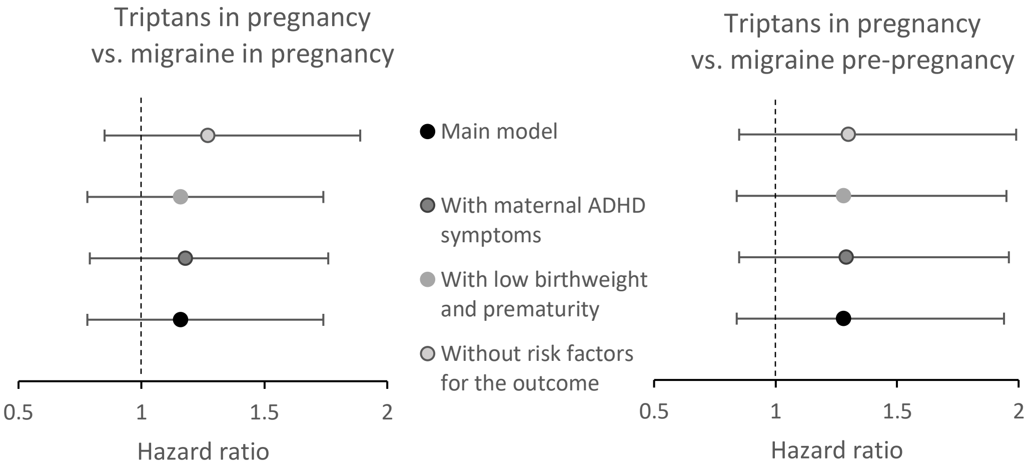

eFigure 4. Comparing the Association Between Prenatal Triptan Exposure and Child ADHD Diagnosis Based on Different Specifications of Inverse Probability of Treatment Weights

© 2022 Harris GM et al. *JAMA Network Open.*

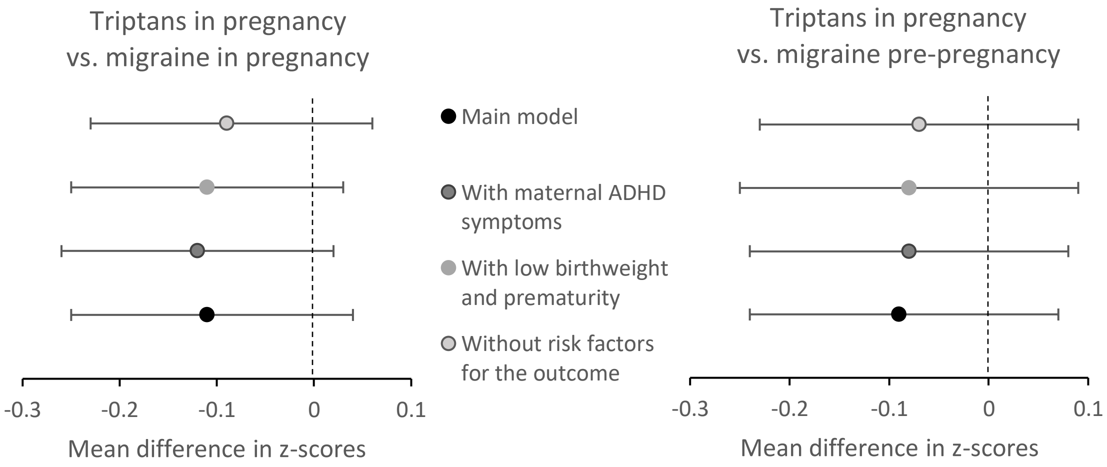

eFigure 5. Comparing the Association Between Prenatal Triptan Exposure and Child ADHD Symptoms Based on Different Specifications of Inverse Probability of Treatment Weights

© 2022 Harris GM et al. *JAMA Network Open.*

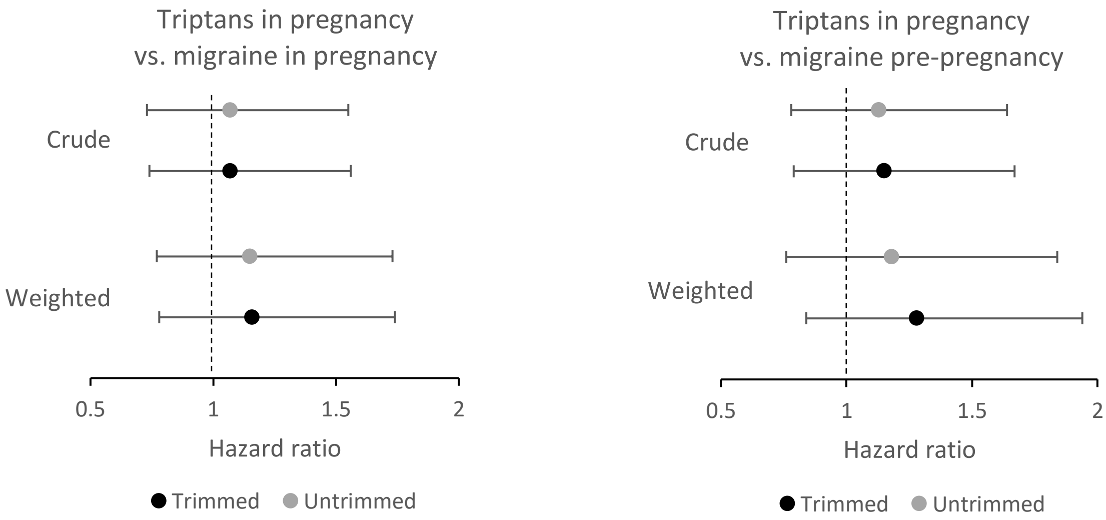

eFigure 6. Comparing the Association Between Prenatal Triptan Exposure and Child ADHD Diagnosis Using Trimmed vs Untrimmed Weights

© 2022 Harris GM et al. *JAMA Network Open.*

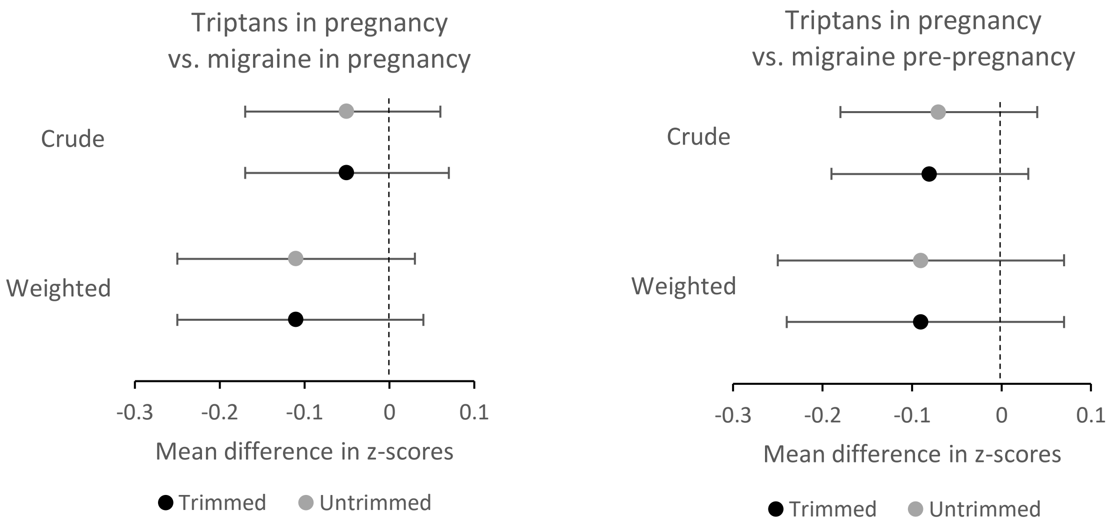

eFigure 7. Comparing the Association Between Prenatal Triptan Exposure and Child ADHD Symptoms Using Trimmed vs Untrimmed Weights

© 2022 Harris GM et al. *JAMA Network Open.*

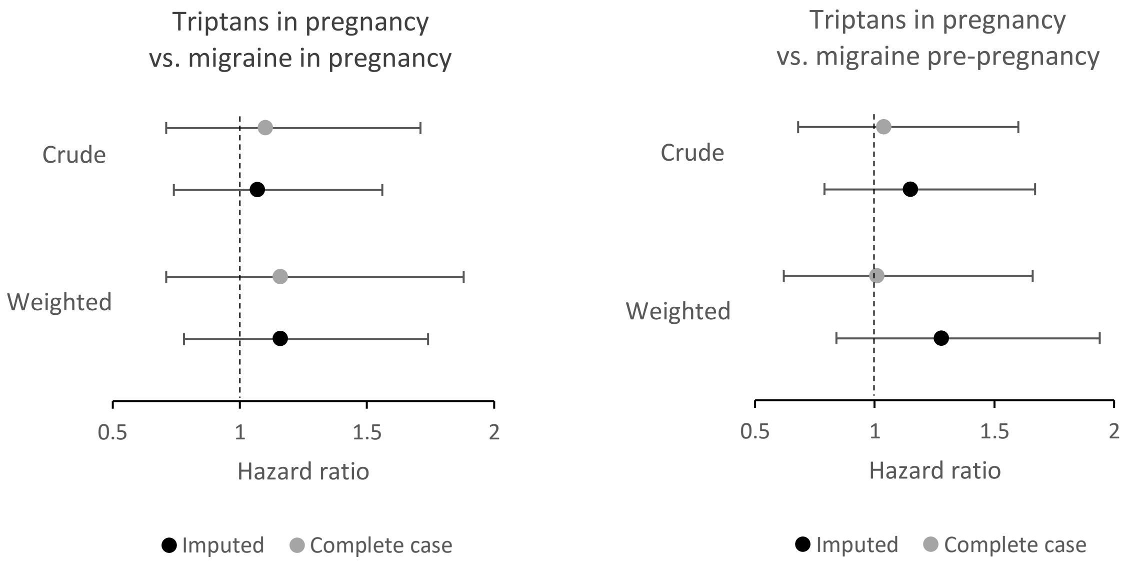

eFigure 8. Comparing the Association Between Prenatal Triptan Exposure and Child ADHD Diagnosis Based on Imputed Data vs Complete Cases

© 2022 Harris GM et al. *JAMA Network Open.*

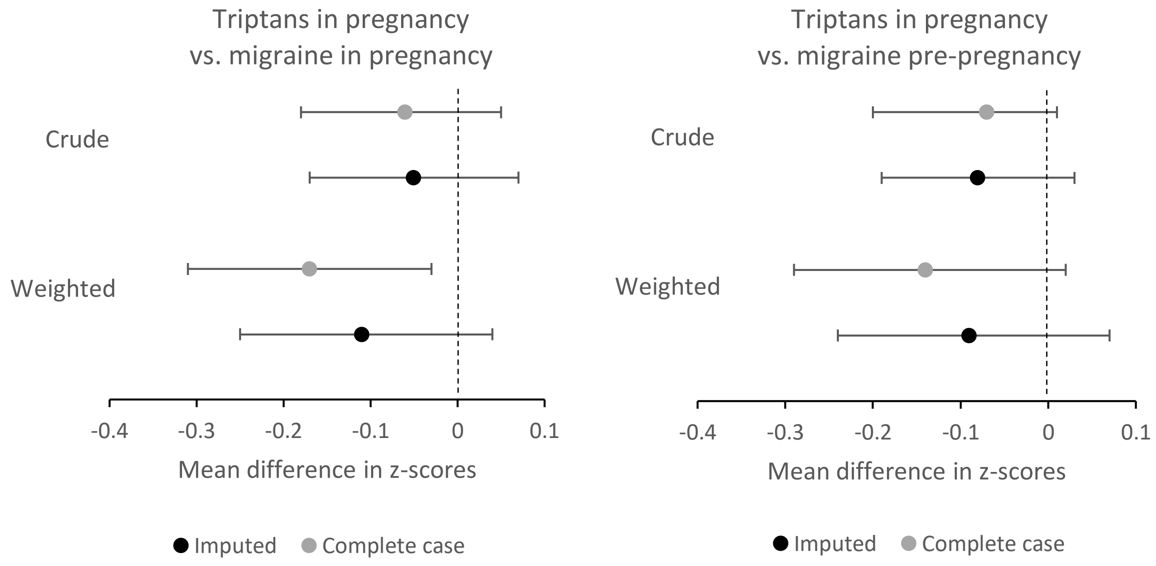

eFigure 9. Comparing the Association Between Prenatal Triptan Exposure and Child ADHD Symptoms Based on Imputed Data vs Complete Cases

© 2022 Harris GM et al. *JAMA Network Open.*

eReferences.

1. Norwegian Mother, Father and Child Cohort Study (MoBa): Questionnaires from MoBa. The Norwegian Institute of Public Health. https://www.fhi.no/en/studies/moba/for-forskere-artikler/questionnaires-from-moba/. Published 2005. Updated 2021. Accessed January 10, 2022.
2. Sterne JA, White IR, Carlin JB, et al. Multiple imputation for missing data in epidemiological and clinical research: potential and pitfalls. *BMJ.* 2009;338:b2393.
3. White IR, Royston P, Wood AM. Multiple imputation using chained equations: Issues and guidance for practice. *Stat Med.* 2011;30(4):377-399.
4. Granger E, Sergeant JC, Lunt M. Avoiding pitfalls when combining multiple imputation and propensity scores. *Stat Med.* 2019;38(26):5120-5132.
5. White IR, Royston P. Imputing missing covariate values for the Cox model. *Stat Med.* 2009;28(15):1982-1998.
6. Sturmer T, Rothman KJ, Avorn J, Glynn RJ. Treatment effects in the presence of unmeasured confounding: dealing with observations in the tails of the propensity score distribution--a simulation study. *Am J Epidemiol.* 2010;172(7):843-854.
7. van Gelder MM, van Rooij IA, de Walle HE, Roeleveld N, Bakker MK. Maternal recall of prescription medication use during pregnancy using a paper-based questionnaire: a validation study in the Netherlands. *Drug Saf.* 2013;36(1):43-54.
8. van Gelder M, Vorstenbosch S, Te Winkel B, van Puijenbroek EP, Roeleveld N. Using Web-Based Questionnaires to Assess Medication Use During Pregnancy: A Validation Study in 2 Prospectively Enrolled Cohorts. *Am J Epidemiol.* 2018;187(2):326-336.
9. Kumar G, Steer RA. Factorial validity of the Conners' Parent Rating Scale-revised: short form with psychiatric outpatients. *J Pers Assess.* 2003;80(3):252-259.
10. Conners CK, Sitarenios G, Parker JD, Epstein JN. The revised Conners' Parent Rating Scale (CPRS-R): factor structure, reliability, and criterion validity. *J Abnorm Child Psychol.* 1998;26(4):257-268.

© 2022 Harris GM et al. *JAMA Network Open.*
